# Understanding the psychosocial determinants of effective disease management in rheumatoid arthritis to prevent persistently active disease: a qualitative study

**DOI:** 10.1136/rmdopen-2024-004104

**Published:** 2024-04-12

**Authors:** Nkasi Stoll, Mrinalini Dey, Sam Norton, Maryam Adas, Ailsa Bosworth, Maya H Buch, Andrew Cope, Heidi Lempp, James Galloway, Elena Nikiphorou

**Affiliations:** 1 Psychological Medicine, King's College London Institute of Psychiatry, Psychology & Neuroscience, London, UK; 2 Centre for Rheumatic Disease, King's College London Faculty of Life Sciences & Medicine, London, UK; 3 Department of Inflammation Biology, King's College London, London, UK; 4 Academic Department of Rheumatology, King's College London, London, UK; 5 School of Immunology & Microbial Sciences, King's College London Faculty of Life Sciences & Medicine, London, UK; 6 National Rheumatoid Arthritis Society, Berkshire, UK; 7 Centre for Musculoskeletal Research, The University of Manchester, Manchester, UK; 8 Centre for Rheumatic Diseases, King's College London, London, UK; 9 School of Medical Education, King's College London, London, UK; 10 Rheumatology Department, King's College Hospital NHS Trust, London, UK

**Keywords:** Rheumatoid Arthritis, Treatment, Qualitative research, Health-Related Quality Of Life, Psychology

## Abstract

**Background:**

According to epidemiological studies, psychosocial factors are known to be associated with disease activity, physical activity, pain, functioning, treatment help-seeking, treatment waiting times and mortality in people with rheumatoid arthritis (RA). Limited qualitative inquiry into the psychosocial factors that add to RA disease burden and potential synergistic interactions with biological parameters makes it difficult to understand patients’ perspectives from the existing literature.

**Aim:**

This study aimed to gather in-depth patient perspectives on psychosocial determinants that drive persistently active disease in RA, to help guide optimal patient care.

**Methods:**

Patient research partners collaborated on the research design and materials. Semistructured interviews and focus groups were conducted online (in 2021) with patients purposively sampled from diverse ethnicities, primary languages, employment status and occupations. Data were analysed using inductive thematic analysis.

**Results:**

45 patients participated across 28 semistructured interviews and three focus groups. Six main themes on psychosocial determinants that may impact RA management were identified: (1) healthcare systems experiences, (2) patient education and health literacy, (3) employment and working conditions, (4) social and familial support, (5) socioeconomic (dis)advantages, and (6) life experiences and well-being practices.

**Conclusion:**

This study emphasises the importance of clinicians working closely with patients and taking a holistic approach to care that incorporates psychosocial factors into assessments, treatment plans and resources. There is an unmet need to understand the relationships between interconnected biopsychosocial factors, and how these may impact on RA management.

**WHAT IS ALREADY KNOWN ON THIS TOPIC**

- Social and psychological factors are associated with disease activity, treatment experiences and outcomes in people with rheumatoid arthritis (RA).

**WHAT THIS STUDY ADDS**

- An in-depth understanding of the complex relationships between interconnected social factors and potential synergistic interactions with biological and psychological parameters that accumulate to impact on the effective disease management in people with RA.

**HOW THIS STUDY MIGHT AFFECT RESEARCH, PRACTICE OR POLICY**

- This study encourages clinicians to work closely with patients towards incorporating psychosocial factors (including, holistic patient education, well-being practices, social care) into assessments, treatment plans and resources to provide more personalised holistic treatment and care that facilitates adherence and improved health outcomes.
- Insights from this study can help inform research to investigate potential interactions between psychosocial, clinical and other patient and disease-related factors that can impact RA management and outcomes.

## Introduction

Rheumatoid arthritis (RA) is a chronic autoimmune condition, characterised by swollen, stiff and painful joints. Over time, RA can damage the joints, cartilage and bone, resulting in long‐term disability. RA is associated with various comorbidities and complications including increased risk of cardiovascular disease, gastrointestinal difficulties, sleep problems, lymphoma and depression.^1–6^ Consequently, mortality in patients with RA is higher than in the general population, especially for those with higher disease activity.^7–9^

RA is treated with conventional synthetic disease-modifying antirheumatic drugs (csDMARDs), biological DMARDs (bDMARDs) or targeted synthetic DMARDs.^10^ Persistently active RA (sometimes referred to as refractory or difficult to treat) is characterised by resistance to multiple therapeutic drugs with active or symptomatic disease.^11–13^

It is increasingly recognised that disease activity in people with RA is impacted not only by biological factors, but also non-biological factors, including psychological and social aspects.^14 15^ Epidemiological studies demonstrate social factors (eg, education, socioeconomic status, or access to local resources and amenities) are associated with increased disease activity, pain, poorer functioning, treatment help-seeking, increased treatment waiting times and mortality.^16–20^ Living in an area with high poverty levels can impact functional disability and disease activity among people with inflammatory arthritis due to the limited availability of resources needed to achieve optimal health.^21 22^ Additionally, adults with RA have a lower likelihood of being employed or remaining in work compared with adults without RA.^23 24^ Those employed face a higher possibility of employment loss when their jobs are physically demanding or have minimal control over job activities, location, hours and pace.^25^

Additionally, psychological factors, including mental health and well-being, can impact pain processing, disease activity and quality of life in patients with RA.^26 27^ Depression and anxiety, as well as psychological distress in people with RA, have been associated with poorer clinical outcomes, decreased levels of physical activity and low treatment adherence.^28–30^ Increasing evidence indicates that addressing psychological aspects together with modifiable social characteristics, that is, psychosocial factors, in the presence of adequate pharmacological therapy, can contribute to improved clinical outcomes for patients with RA.^31 32^

However, existing evidence is largely from observational or small cohort studies which, while useful, cannot identify the more nuanced factors which may be contributing to disease activity in people with persistently active RA. Limited qualitative inquiry into psychosocial factors that add to RA disease burden and potential synergistic interactions with biological parameters makes it difficult to understand patients’ perspectives from the existing literature.

This study aimed to gather in-depth information on psychosocial determinants that drive persistently active disease in RA, using qualitative methodology. The main objective was to provide evidence on how to combine health and social resources with ‘traditional treatments’ (ie, drug therapy) to improve care and inform resource allocation and service redesign, ensuring fair and equal access to all. This study is part of the trajectory of a research programme that aims to inform how to tackle health disparities in persistently active RA.

## Methods

### Design

A qualitative study design was applied and in the reporting of this study, we adhered to the Consolidated Criteria for Reporting Qualitative Research guidelines (online supplemental material 1). Patients either attended a semistructured one-to-one interview or focus group, depending on their preference and availability. The study design and materials (including topic guides) were co-developed during two research consultation meetings with two patient research partners who had lived experience of RA^33^ and based on existing literature.^20 34 35^

10.1136/rmdopen-2024-004104.supp1 Supplementary data

### Participants’ recruitment

Purposive sampling^36^ was used to recruit from rheumatology clinics at a large university hospital that serves a diverse population in London, United Kingdom (UK). Patients who met the following criteria were eligible for participation: (1) minimum age of 18 years, (2) confirmed diagnosis of RA by a consultant rheumatologist, (3) any disease duration or treatment. All patients who met the eligibility criteria were invited via email to complete a sociodemographic questionnaire (eg, age, ethnicity and employment) to ensure the inclusion of a wide range of lived experiences. The sample size (n=45) was guided by data saturation.^37^

### Data collection

Interview and focus group schedule questions elicited participants’ responses about perceived psychosocial barriers and facilitators to effective disease management in RA to pre-empt persistently active disease (online supplemental material 2).

Two online pilot interviews were conducted before the main interviews to assess research burden, timing of data gathering, and the relevance and comprehension of questions.^38^ No changes were made to the schedules. Online interviews and focus groups were conducted in 2021 on Microsoft Teams by an experienced researcher. Interviews lasted on average 30 min and focus groups were on average 90 min. Interviews and focus groups were audio-recorded and transcribed by a postdoctoral qualitative researcher (NS), and personal information and identifiable locations were anonymised. Interpreters were offered for those with self-reported low English-speaking proficiency.

### Data analysis

The researchers followed a step-by-step approach to conducting trustworthy, rigorous and credible thematic analysis suggested by Nowell and colleagues.^39^ Data analysis was led by NS who identified a framework of themes and subthemes,^40^ facilitated using NVivo V.14 software^41^ to manage and organise the data. Analysis began with reading and familiarisation with the data, followed by coding and identifying themes. Differences, similarities, and patterns within and between data sets were recorded in an iterative process. Themes were defined, named and illustrated with vivid examples of data extracts (online supplemental material 3). Themes were reviewed and refined in collaboration with rheumatology clinical-research professionals (EN, MD and SN) during three online consensus meetings. This approach was employed to minimise researcher bias from lone interpretations and personal opinions.^39^

10.1136/rmdopen-2024-004104.supp3 Supplementary data

## Results

### Participant characteristics

45 people participated in the study (n=17 focus group participants, with n=7 in group one and n=5 in groups two and three). Two participants were assisted by an interpreter during their interviews, and all other participants were self-reported fluent in English. Most reported that their RA symptoms were not under control (47%) and they had attended their rheumatology clinic less than 6 months prior to participating in the study (96%). Most were currently receiving csDMARD or bDMARD treatment (62%), and 11% had discontinued treatment. Characteristics of participants are presented in table 1.

**Table 1:** Characteristics and treatment details of participants (n=45)

	n	%
Gender		
Female	38	84
Male	7	16
Ethnicity/nationality		
White/British/European	21	47
Black/African/Caribbean/black British	9	20
Latin American/Hispanic	8	18
Asian/Asian British	7	15
Sexual orientation		
Heterosexual	43	96
Bisexual	1	2
Non-binary	1	2
Age group		
25–34	8	18
35–44	10	22
45–54	15	33
55–74	8	18
75 or older	4	9
Employment status		
Employed full-time	13	29
Employed part-time	7	16
Retired	9	20
Student	2	4
Unemployed	14	31
Occupation		
Education profession	8	18
Human health and social work activities	7	16
Construction	5	11
Retail and sales	4	9
Manufacturing	1	2
Not applicable/unknown	20	44
Main language spoken or written at home		
English	28	62
Spanish	8	18
Hindi	4	9
Cantonese	2	4
Polish	2	4
French	1	2
Patient perception of disease activity		
Not under control	21	47
Under control	14	31
Unsure	10	22
Last disease assessment by a clinician		
Less than 6 months ago	43	96
6 months–1 year ago	2	4

### Study findings

**Figure 1:**
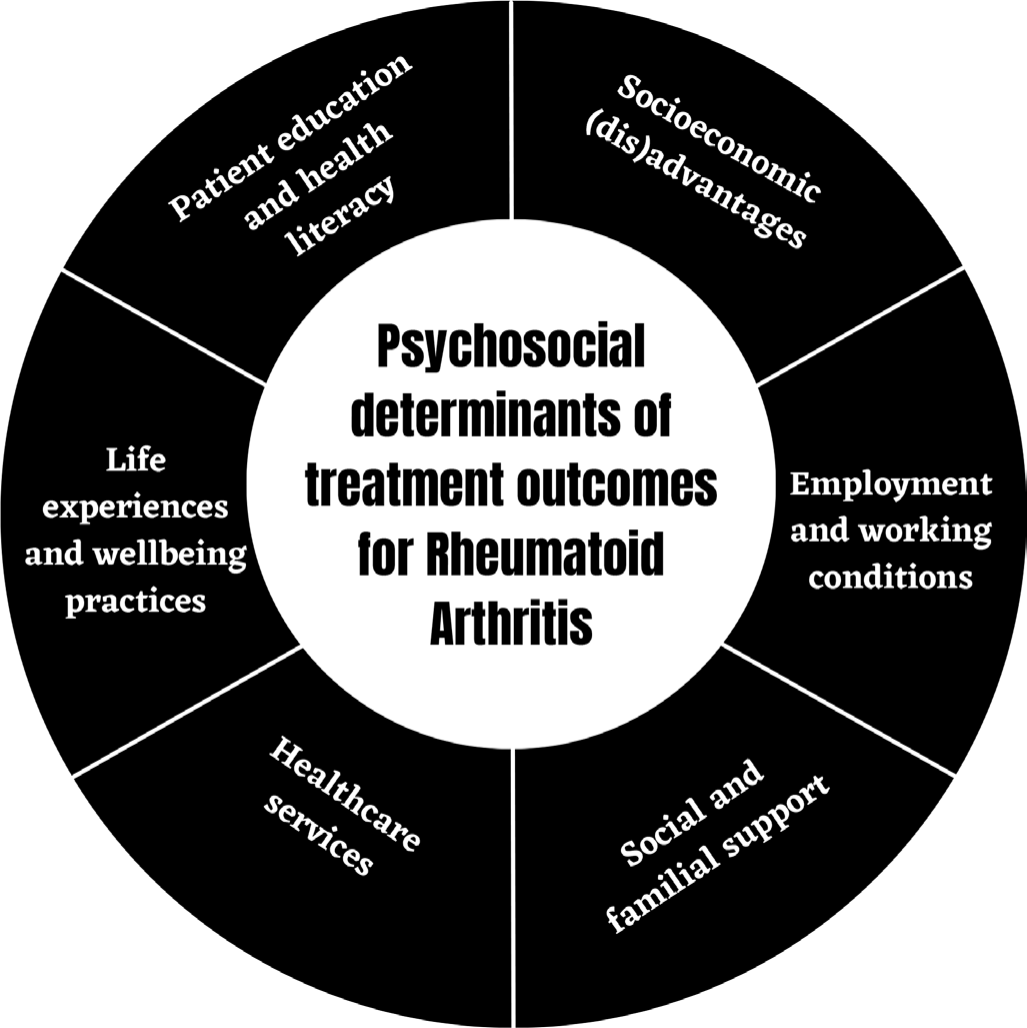
Psychosocial determinants of effective disease management in rheumatoid arthritis to prevent persistently active disease according to patient perspectives.

Six psychosocial factors for effective RA management to pre-empt persistently active disease (figure 1) were derived with 12 subthemes (online supplemental material 4).

10.1136/rmdopen-2024-004104.supp4 Supplementary data

### Life experiences and well-being practices

#### Stressful life experiences and poor well-being

Many participants spoke about struggling to balance competing social roles and responsibilities while managing their RA. These daily life stressors included caring for their children, working and progressing in their careers, completing housework, maintaining a romantic relationship and social life, engaging in mental and physical well-being practices (eg, movement and mindfulness) for their health, and studying. Participants described feeling unable to cope with the stress, sometimes leaving them bedridden with physical and mental exhaustion, especially those who did not feel supported by their partners, family or friends.

With stress around the house, taking care of the children, [managing] the disease, and I don’t have any support from my husband, it can make me depressed. (Latin American/Hispanic woman, 35–44)

Participants also disclosed that stressful life events and experiences exacerbated their RA symptoms and impacted their ability to adhere to their RA treatment. Some adverse events and experiences mentioned were the death of loved ones, divorce, drug or alcohol misuse and moving home abruptly. Due to these stressors, individuals described various disruptions in their ability to manage their condition, such as forgetting to take their medication, not attending clinical appointments, and de-prioritising physical and mental well-being practices.

I used class A drugs [because they] have added vitamins, like vitamin D, that are vital for maintaining my joint health so I used cocaine to try and get through a flare-up and [to mentally] cope with my new [RA] diagnosis…but in the end it made [my RA symptoms] worse. (white woman, 35–44)

Several described feeling symptoms of depression, anxiety and poor mental well-being (including low mood, sleep problems, panic attacks, hopelessness, anger and eating disturbances) throughout their lives due to compounding social demands and complications of RA. Many expressed having frequent negative and anxious thoughts about the progression of their RA, loss of independence and individuality, and early mortality. Experiencing debilitating pain, learning that treatment was ineffective, and the onset of complications and comorbid disorders were particular situations that triggered these mental symptoms.

All the tears are coming. Anxiety, panic attacks, depression, many negative feelings are coming, and I feel frustrated… Sometimes I am in so much pain that my mood is really low. Sometimes I feel that it is better to be dying instead of living [with a disability] but I don’t want to die. (Asian woman, 45–54)

During poor mental health episodes, participants said that they did not maintain personal hygiene, attend clinical appointments or take their RA medication. They described engaging in behaviours that would exacerbate their RA symptoms, including eating high-inflammatory foods, ceasing physical and mental wellness practices, and isolating themselves from friends and family. A couple of participants said they engaged in risky alcohol misuse despite knowing that this might impact the effectiveness of their RA medication. These behaviours were said to exacerbate and maintain RA flare-ups, and inevitably worsen their mental health.

When my mood is low, I crave a lot more sugar [to] help lift my energy, but then that leads to a crash and triggers my RA. So, I am going around and around in a cycle looking for ways to improve my [mental and physical] health, but it is harder to [engage in] healthy [RA management] as you have less energy and less ability to stick [to your treatment] because you are focusing on the pain and sadness more. (white woman, 25–34)

#### Mental and physical well-being practices

Practising daily ‘positive thinking’ was used by participants to cope with their RA symptoms and prognosis, and helps them stay focused on managing their RA. They described using humour, practising positive self-talk, surrounding themselves with positive people and engaging in mindfulness.

Feeling positive that things will be better one day helps me to take my medication and manage my illness. Hope is important that the treatment will help. (Latin American/Hispanic man, 45–54)

Maintaining hobbies, interests and movement that instilled joy was reported to help distract from physical and mental pain, and feeling motivated to engage with treatment plans. Effective well-being activities and practices mentioned included knitting, dancing, singing, gardening, swimming, cycling, yoga, reading, mindfulness meditation, walking, cooking, playing board games, and attending sports and art events.

Having things to look forward to helps me live with RA, like holidays or dancing with friends, or singing. You can overcome the pain by feeling better about yourself and your life. (Black woman, 25–34)

Several used complementary medicine and other pain relief alongside their prescribed RA medical treatment. Regular use of RA medication was perceived to ‘have a traumatising effect on the body’ and using non-medical treatments was explained as to allow participants to feel in control of their care. The non-medical treatments mentioned were acupuncture, massages, homeopathy, herbal medications, ozone sauna therapy, red light therapy and pulsed electromagnetic field therapy.

I used plant-based medication for RA, and I think that’s been very good to heal me…I don’t just want to accept my [medical] treatment with no control and no power over what goes in my body. (white woman, 35–44)

### Socioeconomic (dis)advantages

#### Socioeconomic disadvantages

Those who had to climb stairs to access their property and inside the home described feeling physically exhausted and frustrated daily. Some participants reported that their landlords refused to upkeep their homes to an appropriate standard (eg, mould and damp) or install disability adaptions. Inadequate housing conditions were mentioned to cause RA flare-ups, comorbid health conditions and poor mental health, and hindered their ability to care for themselves.

[Stairs] can become a big barrier [to] getting through my day, and it just makes RA a lot worse, because I am physically exhausted…I wish I could afford a bungalow. (white woman, 65–74)

Many spoke about not being able to afford private transportation (ie, cars or taxis) to their RA appointments, which often impacted on their ability to attend their appointments on time. Those who had to use public transport (ie, buses or trains) described long waiting times, pain and RA flare-ups due to having to walk, sit or stand in uncomfortable conditions for prolonged periods.

The [hospital outpatients] transport does not allow family to come… Sometimes the transport comes late, and they offer a minicab, but I can’t afford it and I need help getting in the cab and [transport staff] can’t help me. This causes a problem at the hospital [because I am late], so I have to wait [longer] to see the doctor. (black woman, 45–54)

Living near their clinic was said to facilitate participants’ appointment attendance and promptness, and minimise travel stress and burden on their physical health.

#### Socioeconomic privileges

Financial advantages were reported to minimise stress and improve access to quality treatment, which impacted disease activity. Privileges included financial support from their families to pay for costly RA care (eg, prescriptions, or hot and cold treatments). Lack of financial access to meal preparation services, ‘healthy’ anti-inflammatory ingredients or a kitchen with accessible cooking equipment impacted their ability to manage their RA with nutritious foods.

Financially being able to cover the cost of greens and veg[etables] you have to be able to afford nutritious and [anti-inflammatory] meals, and bring [nutritious food] home, and actually being able to prepare those meals as well in your kitchen. (white woman, 25–34)

Cold and damp weather was reported to trigger many participants’ RA flare-ups. However, those who could afford to keep their heating on continuously, or travel to a warm country during UK winters, described feeling better able to manage their RA. Participants also noted that access to a private garden or local green space with amenities (eg, allotment, café, walking paths) helped them stay active.

We are lucky to have a big, beautiful park close by…I find being outside in nature helps me a lot, because it encourages me to walk in a relaxing way, which reduces the [RA] disease activity. (white woman, 45–54)

### Employment and working conditions

#### Issues with employment and working conditions

Numerous participants recalled employers being unaccommodating of their hospital appointments and/or sick leave requests, which impacted their medical care. Many also described having low job autonomy over work location or hours, and no access to assistive technology or equipment (eg, ergonomic mouse or chair).

I often had flare-ups because I was on my feet or sitting in a chair all day and it felt like my whole body was on fire in the morning. (white woman, 45–54)

Participants described experiencing debilitating RA flare-ups before, during or after work due to their jobs being physically demanding (ie, manual labour, standing or walking continuously, disrupted sleep) and emotionally taxing (eg, working under constant pressure or caring for others). They spoke about the ‘physical cost’ of having to carry out strenuous routine physical activities (eg, travelling on public transport and completing personal hygiene practices in the mornings when RA pain is at its peak).

My flare-ups happen because of the pressure of work and having to travel back and forth. I would attend blood test appointments and I’d be waiting there for hours and hours thinking about work. I used to wake up and start work earlier and come back and work later, which was stressful on my body. (Asian woman, 25–34)

Over the years, these work stresses were said to cause increasing pain, anxiety, low mood, and chronic stress symptoms which led to repeated lateness and absence from work, low job motivation, and poor work performance. Consequently, many described being unfairly disciplined or dismissed from work by unsympathetic employers.

I was in hospital a lot, and I lost my job within weeks of a promotion which was very frustrating…I was in a really bad place, [after losing my job] is when my RA [symptoms] started to spiral. I spent 5 weeks indoors; I didn’t leave the house. (white woman, 45–54)

#### Supportive employment and working conditions

Many participants mentioned that having a stable and sufficient income meant they were able to afford vital healthy practices (eg, expensive healthy foods, private transportation, house cleaning services, disability-friendly holidays for respite). ‘Supportive’ and ‘compassionate’ employers and colleagues were praised for helping participants feel valued, respected and capable at work.

[My colleagues] ask me frequently how I feel and if everything is all right and how is my RA, and if my medication is working better. They make me feel hopeful. (Latin American/Hispanic woman, 35–44)

Another example of compassion was a participant’s employer sanctioning their sick leave without recording their absence to Human Resources to ensure they did not exhaust their leave allowance.

Being able to work from home, with flexible hours, helped participants adhere to their treatment plans and recover from flare-ups. Benefits included being able to better manage their pain symptoms by controlling the temperature in their home workspaces, take necessary rest breaks in bed, have easy access to their medications and home remedies. Other helpful work accommodations included having a budget for private transportation to and from work, longer rest breaks, emergency work cover and reduced work duties.

[Mental] stress makes RA worse, so having these work provisions and an understanding [work] environment allows me to relax and not worry about not being able to do my job or go to [RA] appointments. (white woman, 35–44)

Some patients worked or volunteered to distract themselves from physical pain and emotional distress. All volunteers sought out work with children and families, to ‘make a difference in the lives of others’ and feel less lonely. This was said to boost their quality of life and self-image.

Volunteering really helps, gives me some positivity, [and] something to do and takes my mind off the pain. [This] helps because there is a tendency to wallow in the pain sometimes. (white woman, 75 or older)

### Healthcare services

#### Adverse experiences of treatment and severity of illness

Participants discussed numerous structural challenges to attending their clinical appointments or receiving appropriate treatment and care. They mentioned long waiting times between referrals and their first appointment with a specialist (up to 6 months), appointment delays (over 2 hours) and living a considerable distance away from the nearest rheumatology clinic. One individual described being left without care for over a year because their medical records were ‘lost in the system’, which increased their disease activity.

Many recalled that during the COVID-19 pandemic, their disease activity increased because they were unable to access their medical treatments or appointments. For some, this experience had a long-term impact on their RA progression and prognosis.

As a result [of COVID-19] I suffered a lot with inflammation, and I was in pain. If I had my treatment on time, I wouldn’t have so much damage in my bones and I wouldn’t have had to have surgery and the mobility wouldn’t be so bad. (Asian woman, 65–74)

Some clinicians were described as ‘aggressive’, ‘patronising’ and ‘impatient’, which prevented participants from attending their appointments, being honest about their symptoms or advocating for treatment changes.

Another barrier for some participants was a lack of interpreters available at their RA clinics, due to cost constraints and lack of diversity of staff. This situation impeded upon some patients’ ability that their specialists had empathy, mutual trust and care towards them. One participant’s friend and interpreter said:

[Participant] was missing a couple of treatments because I, [the participant’s friend], was on holiday, and the hospital did not provide an interpreter for him. [Participant] was unwell and he was trying to find one person to help him with his English but couldn’t, so he suffered. (Latin American man, 35–44)

Easy and early access to effective RA medication, with minimal side effects, was said to facilitate treatment adherence. However, many participants reported various medication side effects and complications throughout their treatment journey (eg, headaches, liver issues, eye problems, allergic reactions, nausea, significant weight gain, osteoporosis and anaemia).

I have been in and out and in and out of hospital trying every [RA] drug there is. Now I have all these other health problems…I just want to stop taking the drugs. (white man, 45–54)

Overall, experiencing unbearable pain due to RA flare-ups was cited as the main reason participants failed to adhere to their correct medication dosage, did not attend their clinical appointments, or stopped engaging in physical and mental well-being practices.

I would rather take more [medication] so that my pain can go away or that I can die. That has come across my mind, [to end my life], a few times when I am in pain. (Asian woman, 45–54)

#### Effective treatment and care

All valued having knowledgeable, competent and friendly RA clinicians. These qualities facilitated treatment adherence and clinician–patient alliance. They praised RA clinics that offered an option for face-to-face, telephone or video appointments. Some also appreciated having text and call reminders from hospital staff to attend their RA appointments. These services were said to help participants gain access to relevant information.

I like going to the hospital because I am confident my doctor is knowledgeable about RA. [My RA clinicians] are human beings, are understanding, patient, listen and sometimes say “Okay, no worries.” (white woman, 25–34)

Patients also described relying on numerous aids and adaptions around their homes to help them manage their RA, including medicine dispensers, assistive kitchen utensils, home adaptations and mobility aids. These gadgets and modifications were said to improve their quality of life and independence.

Having [aids and adaptions] means I can cope, I can take my medication, hold a fork, and I don’t need someone to cut up my food [or food] to be fed to me. [Aids and adaptions] are empowering [because] you can do things for yourself. (white woman, 35–44)

### Holistic RA information and knowledge

#### Gaps in education and knowledge

Some participants reported that rheumatologists often failed to provide sufficient and necessary information about the prescribed tests, examinations and treatments for RA. Treatment adherence was said to be impacted by a lack of knowledge from medical staff on how RA medications work, possible side effects and when to take treatment, especially when patients were also prescribed medication for other life-threatening health conditions.

I had a heart attack recently. I asked the consultant cardiologist when I saw him [after surgery] if it was okay to take [my biologic] now and he did not have a clue what the biologic was…I have been too scared to take it [biologics] since, that is my own fault. (white man, 65–74)

Many expressed frustration that their rheumatology specialists did not educate them about holistic non-medical RA symptom triggers and treatments. Engagement (or lack of) in the following lifestyle changes was said to impact RA symptoms and form an important part of their RA management: anti-inflammatory foods (eg, ginger or omega-3), herbal remedies (eg, green tea), movement and exercise (eg, weight and strength training or yoga), and natural treatments for pain reduction (eg, aromatherapy, meditation or essential oils).

My RA is treated with medication, but no [medical professional] has ever spoken to me of the more holistic approach, about diet, lifestyle and I think that is something that has been missing in the education received about RA. (white woman, 35–44)

Some non-native English speakers expressed their disappointment that their hospitals did not provide information about RA in their native language, which caused confusion about the scope of their condition, and how and when to take medications.

It would help to have written information in Spanish about [my] treatment to prepare [myself] and understand more clearly about what medication [I am] taking. [This] would [help me feel] more confident about taking the medication. (Latin American/Hispanic woman, 65–74)

A few mentioned that they were ill-prepared to manage the psychological impact of living with RA. They wanted more information on how to stay motivated to adhere to their RA medical treatment despite adverse side effects and how to cope with changes to their body, interpersonal relationships, and physical and psychological well-being.

I wanted my doctor to give me warnings about ‘this is what to expect with RA, be gentle with yourself and allow yourself some space to adjust mentally to the dangers that are going to come’. [Patient education] should be more about preparing someone for this phase of their life. (black woman, 25–34)

#### Health literacy and holistic knowledge

Participants praised clinics that provided access to impromptu RA advice via email, helplines, text or apps. They valued having multiple opportunities to discuss the personal causes, treatments, possible side effects, potential triggers for flare-ups and prognosis for their RA with their specialists. Many expressed that this access to medical information helped develop trust in their doctors’ advice, boost their confidence to adhere to their treatment plan and provide hope that treatment might be successful. Participants also appreciated having educational leaflets, videos and information on the hospital websites that provided novel information on RA and tips on how to accomplish daily tasks. Tips included how to open a car door, to organise and remember to take medication on time, or how to chop food.

One consultant explained the markers because I wanted to monitor the food and lifestyle changes that I’m trying, so when I do the blood test I can see if [biomarkers] going up or down…[The consultant] told me what to look for in the blood test… The information gives me clarity and makes me feel confident. (Asian woman, 35–44)

For many, their holistic RA knowledge came from watching social media awareness posts, reading online blogs written by specialists, carers and patients, or receiving advice from friends or family who have inflammatory diseases. Many also used charities (eg, National Rheumatoid Arthritis Society and Versus Arthritis) to gain holistic information and knowledge about RA.

### Social and familial support

#### Limited or no access to social and familial support

Due to the severity of their RA symptoms, some participants reported being excluded from social activities due to family, friends and colleagues holding ableist beliefs about RA and disability. This left them feeling socially isolated.

There’s a bad attitude towards people with a disability. [People] are very quick to judge but not to understand and empathise. It makes me feel alone and makes me want to stay indoors and not go out, it affects the actual disease itself because it makes you feel tired. (white woman, 35–44)

#### Social and familial support

In contrast, patients who had social connections that were knowledgeable about their RA triggers and management helped motivate them to adhere to their treatment and care, distracted them from their RA symptoms and boosted their mood.

My friends have knowledge [about] how I control my disease and the triggers and are understanding. They look out for me and look after me when I am not doing well and can’t look after myself. (Latin American/Hispanic man, 25–34)

Online or in-person patient-led groups provided important spaces for participants to share advice on how to manage flare-ups and daily tasks. Patients also motivated each other to adhere to treatment and healthy lifestyle choices. These groups were said to help them feel less alone.

I talk to other people around the world who also suffer from RA, we all have the same thing, we all understand each other and how we feel. (white woman, 45–54)

Several stated that engaging in religious and spiritual practices (eg, prayer, reading holy texts, attending places of worship or listening to gospel music) helped with pain acceptance, stress reduction and social connections.

I have a lot of people [from the church congregation], who visit or if I need things are very caring and provide for me… [my religious faith] does give me a lot of strength and confidence. (black woman, 75 or older)

Participants recounted numerous occasions when during an RA flare-up, their nuclear and extended families carried out caring responsibilities. These included domestic tasks, physical and personal care, childcare and psychological support. Fear of being a ‘physical and emotional burden’ on their families was cited as an important reason participants adhered to their RA treatment and care.

My kids are my motivation… If my disease is worse and I am in pain, then I feel I will be a terrible mother. They are small, they want to play, I want to do things with them [so] my body needs to work. (Asian woman, 45–54)

## Discussion

This study provides in-depth patients’ perspectives on the psychosocial determinants of effective disease management in RA to pre-empt persistently active disease. The findings align with previous studies, positing that social factors, such as education, employment, healthcare and social resources, play an important role in RA disease activity and management.^17 26 34 42^ The added value of our study is the exploration of *how* these social factors may interact with psychological and personal contexts to compound the impact of RA disease activity and management.

In our study, RA management was affected by health factors, specifically, patients’ perceived severity of symptoms, and access to effective personalised and holistic care, treatment, and RA education. According to our findings, these factors impacted and were impacted by compounding social stressors, such as stressful life experiences and daily responsibilities. In turn, social stressors and health factors shaped and were shaped by an individual’s psychological factors (specifically mental well-being and well-being practices) and personal factors, such as financial privileges, social and familial support. The interplay between these psychosocial factors influenced patients’ perceived treatment experiences and outcomes, personal health literacy, well-being and quality of life (online supplemental material 5).

10.1136/rmdopen-2024-004104.supp5 Supplementary data

The study findings indicate several implications and recommendations. First, with so many determinants influencing RA management and outcomes, a ‘one-size-fits-all’ approach would not be sufficient to address the underlying multiple needs of patients. Participants expressed the desire to talk regularly about their housing issues, relationship problems, poor mental health, work stresses and dietary changes with their clinicians. Clinicians might want to make it a priority to routinely discuss how these circumstances impact patients’ RA symptoms, treatment efficacy and prognosis. Simultaneously, improving referral and signposting pathways between medical teams, social care and psychological services might provide more accessible and effective support for patients.

Potential solutions to improve patient outcomes could be ensuring written and video educational materials are easy to comprehend, culturally relevant, available in the primary languages spoken by patients, and include medical and psychosocial information. Also, hospitals could co-design patient-led RA peer support groups that encourage social connectedness and sharing of effective ways to cope with physical and mental RA symptoms. Furthermore, providing family interventions that focus on holistic care and well-being practices could be useful in improving family dynamics, RA symptoms, treatment adherence and outcomes. Any initiatives might benefit from collaboration and incorporation of work by patient-centred RA charities and societies.^43^ These solutions might help ensure information is accessible to people with different levels of health literacy.

Our study findings complement existing research calling for improvements in social care policies and services to incorporate integrated social, psychological and healthcare models for RA.^44 45^ These improvements need to be based on the understanding that numerous individuals and institutions are responsible for the quality of life, disease management and outcomes of patients with RA. Future work needs to focus on examining the role of, and intricate relationship between psychosocial factors, using large-scale epidemiological data. The findings of our work will be a starting point to inform the development of a national survey to obtain greater insights into biopsychosocial interactions and their impact on outcomes in patients living with RA and other long-term, inflammatory joint diseases.

The study findings, implications and recommendations need to be considered in the context of several strengths and limitations. First, data collection occurred in 2021 during the coronavirus pandemic which was marked by severe disruptions in healthcare, economic instability, social restrictions and health and well-being concerns in the UK, especially for people with disabilities.^27 46^ This context gave us unique insight into how patients manage their RA during a global crisis. This study was strengthened by the involvement of patient research partners, which is a recommended strategy for improving the quality and relevance of the research process and findings.^33^ Furthermore, the team recruited a diverse sample by ensuring accommodations were met, which included interpreters, providing all information in plain language, conducting interviews online and providing study information on an internet site that was compatible with screen readers.^47^ However, more representation from under-represented and marginalised identities (eg, racial, ethnic or cultural minority groups, and, sexual and gender minority groups) could have revealed further sociocultural differences and improved the transferability of the findings.^48 49^ Nevertheless, the consensus and robustness of participants’ perspectives, who represented different ethnicities, languages, ages, employment statuses and occupations, indicated that the study findings may be relevant to diverse populations with RA.

In conclusion, this study adds to the evidence that beyond biological factors, psychosocial factors also play a role in health and RA management^50 51^ and highlights the value and need for clinicians to ask, support, signpost and record psychosocial contexts, and holistic care and treatment. Taking a collaborative and integrated biopsychosocial care approach can enhance patient care and experience, minimising the risk of treatment failure and maximising health potential for all, irrespective of social background.

10.1136/rmdopen-2024-004104.supp2 Supplementary data

## Data Availability

All data relevant to the study are included in the article or uploaded as supplemental information.
