# Supplementary material for: Understanding the psychosocial determinants of effective disease management in rheumatoid arthritis to prevent persistently active disease: a qualitative study
Source: RMD Open. 2024 Apr 12;10(2):e004104. doi: 10.1136/rmdopen-2024-004104 (PMC11029421; doi:10.1136/rmdopen-2024-004104)
Supplement: Supplementary data [file rmdopen-2024-004104supp001.pdf]

Understanding the psychosocial determinants of effective disease management in Rheumatoid Arthritis to prevent persistently active disease: A qualitative study

Supplementary Material 1: Consolidated criteria for reporting qualitative studies (COREQ): 32-item checklist

Developed from:
Tong A, Sainsbury P, Craig J. Consolidated criteria for reporting qualitative research (COREQ): a 32-item checklist for interviews and focus groups. *International Journal for Quality in Health Care*. 2007. Volume 19, Number 6: pp. 349 – 357

**No. Item**	**Guide questions/description**	**Reported on Page #**
**Domain 1: Research team and reflexivity**		
Personal Characteristics		
1. Interviewer/facilitator	Which author/s conducted the interview or focus group?	Page 5
2. Credentials	What were the researcher's credentials? E.g. PhD, MD	Page 5
3. Occupation	What was their occupation at the time of the study?	Page 5
4. Gender	Was the researcher male or female?	N/A
5. Experience and training	What experience or training did the researcher have?	Page 5
Relationship with participants		
6. Relationship established	Was a relationship established prior to study commencement?	Supplementary material 2
7. Participant knowledge of the interviewer	What did the participants know about the researcher? e.g. personal goals, reasons for doing the research	Supplementary material 2
8. Interviewer characteristics	What characteristics were reported about the interviewer/facilitator? e.g. Bias, assumptions, reasons and interests in the research topic	Page 5

**Domain 2: study design**		
Theoretical framework		
9. Methodological orientation and Theory	What methodological orientation was stated to underpin the study? e.g. grounded theory, discourse analysis, ethnography, phenomenology, content analysis	Pages 4-5
Participant selection		
10. Sampling	How were participants selected? e.g. purposive, convenience, consecutive, snowball	Page 4-5
11. Method of approach	How were participants approached? e.g. face-to-face, telephone, mail, email	Page 5
12. Sample size	How many participants were in the study?	Page 5
13. Non-participation	How many people refused to participate or dropped out? Reasons?	Page 5-6
Setting		
14. Setting of data collection	Where was the data collected? e.g. home, clinic, workplace	Page 5
15. Presence of non-participants	Was anyone else present besides the participants and researchers?	Page 5
16. Description of sample	What are the important characteristics of the sample? e.g. demographic data, date	Page 5-6
Data collection		
17. Interview guide	Were questions, prompts, guides provided by the authors? Was it pilot tested?	Page 5 and Supplementary Material 2
18. Repeat interviews	Were repeat interviews carried out? If yes, how many?	No
19. Audio/visual recording	Did the research use audio or visual recording to collect the data?	Page 5
20. Field notes	Were field notes made during and/or after the interview or focus group?	Page 5
21. Duration	What was the duration of the interviews or focus group?	Page 5
22. Data saturation	Was data saturation discussed?	Page 5
23. Transcripts returned	Were transcripts returned to participants for comment and/or correction?	No
**Domain 3: analysis and findings**		
Data analysis		

24. Number of data coders	How many data coders coded the data?	Page 5
25. Description of the coding tree	Did authors provide a description of the coding tree?	Supplementary Material 4
26. Derivation of themes	Were themes identified in advance or derived from the data?	Page 5
27. Software	What software, if applicable, was used to manage the data?	Page 5
28. Participant checking	Did participants provide feedback on the findings?	No
Reporting		
29. Quotations presented	Were participant quotations presented to illustrate the themes/findings? Was each quotation identified? e.g. participant number	Pages 8-19 Supplementary Material 3
30. Data and findings consistent	Was there consistency between the data presented and the findings?	Pages 8-19
31. Clarity of major themes	Were major themes clearly presented in the findings?	Pages 8-19 Supplementary Material 3
32. Clarity of minor themes	Is there a description of diverse cases or discussion of minor themes?	Pages 8-19
