# Supplementary material for: Understanding the psychosocial determinants of effective disease management in rheumatoid arthritis to prevent persistently active disease: a qualitative study
Source: RMD Open. 2024 Apr 12;10(2):e004104. doi: 10.1136/rmdopen-2024-004104 (PMC11029421; doi:10.1136/rmdopen-2024-004104)
Supplement: Supplementary data [file rmdopen-2024-004104supp003.pdf]

Understanding the psychosocial determinants of effective disease management in Rheumatoid Arthritis to prevent persistently active disease: A qualitative study

Supplementary Material 3: **Additional illustrative quotes**

Main theme	***Additional illustrative quotes***
**Life experiences and wellbeing practices**	"When I was playing with my kids and she [participant's child] clashed with one of my hands and my hand is painful and it still is, even after three days…It hurts me that I can't play with my kids sometimes".<br><br>"If I do things that I know will destroy my joints, my RA becomes more active… if I buy too many things for the house and carry the bags it affects my joints and causes pain. Often it's things it's hard for you, but you know you have to do for yourself and your family".<br><br>"Having a structure and a routine like getting up the same time every day to take your medication, and showering, eating and sleeping routine helps with RA treatment. If I'm not engaging in the normal everyday routines then I don't engage in my treatment because it's very easy to get complacent about the importance of taking your medication".
**Socioeconomic (dis)advantages**	"I went to a conference…and it was like a bolt of lightning from the sky that I sat next to [the Chief Executive of Hospital in London where the participant is currently being treated for Rheumatoid Arthritis]… she gave me [my current consultant's] email address and telephone number. Myself and my husband often comment that if it had not been for [meeting the chief executive at the conference] then I don't know whether I would have survived".<br><br>"Not having a helper or carer, or having the support of someone cooking those nutritious meals for you…can make [Rheumatoid Arthritis] a lot worse, and you did end up having the processed foods, because they are a bit easier, but [eating processed foods] keeps the flare up going for longer".<br><br>"Having money and access to a car help me attend clinical appointments. I don't think I would be able to attend if I didn't have a car. The times when I haven't had money to travel to the appointments have meant I missed them. I would get the train but they are a struggle and I have to take hygiene into account, because I do worry about that".
**Employment and working conditions**	"I had to give that up a job as a sales assistant, because I was crawling out of work, and the next morning, I couldn't get out of bed. The job wasn't helping, [working as a sales assistant] was physically demanding, as you are on your feet all day for eight hours. And you are doing deliveries and pulling things out of bags, and holding them up, and running around the shop [which] caused pain and swollen joints".<br><br>"When I was working when I had appointments I told my managers that I was ill so that I could attend my appointments but they would look at me with a bad face and tell me this is a business and would ask me to change the appointment date or time".

	"I have a brilliant team [of work colleagues] at my work. My line manager understands what is going on with [my Rheumatoid Arthritis] and that is very helpful. I am not going into work stressed, I am not being given [work] tasks that I cannot handle, because I am not physically capable to perform it…I feel safe at work".
**Healthcare services**	"Some doctors who are not good and are too mechanical and they get rid of the patient too quickly and it's not nice and we suffer… some doctors are not good with us, they are not polite, they are not understanding, they are not approachable. We are already suffering and with doctors like this we are suffering more".<br><br>"Access to supportive doctors and nurses has being really helpful when I've needed to check-in. My rheumatologist was really good [when] I was a teenager and [the rheumatologist] would take social things into consideration, not just giving me the social support but also talking about things like if I ever wanted to get pregnant or if I was drinking alcohol how it would affect me. There was a lot of support there".
**Holistic RA information and knowledge**	"I haven't learnt RA through medical professionals, I've learnt about rheumatoid arthritis through experiencing it. I'm never gonna understand it from a science level because I'm not that way inclined".<br><br>"I have seen certain articles that assume that certain foods…are not good for people with Rheumatoid Arthritis. I have to make sure that my diet is rich in supplements and that I have the right balance of everything. So, having that additional information [on diet] from your consultant would really help".<br><br>"Asking questions comfortably to my doctor has also helped a lot with my treatment, at first I didn't want to take medications but over time and with research I understand and I take my treatment. I talk to people in the medical field more I attend seminars and conferences to learn more about RA and now I am more comfortable with my RA".
**Social and familial support**	"I've gotten used to people not understanding [Rheumatoid Arthritis] before with my family I was like 'this hurts, that hurts' and they were like oh 'why don't you try that, why don't you do this' and now I don't bother talking to them about my [Rheumatoid Arthritis]. They don't understand how I feel".<br><br>"If you feel [socially] isolated then the Rheumatoid arthritis feels bigger and more scary than it is".<br><br>"I take comfort from home, my family, my friends and my pets. If you feel happy it's almost like adrenaline so the pain is not as severe psychologically".
