# Supplementary material for: Understanding the psychosocial determinants of effective disease management in rheumatoid arthritis to prevent persistently active disease: a qualitative study
Source: RMD Open. 2024 Apr 12;10(2):e004104. doi: 10.1136/rmdopen-2024-004104 (PMC11029421; doi:10.1136/rmdopen-2024-004104)
Supplement: Supplementary data [file rmdopen-2024-004104supp004.pdf]

Understanding the psychosocial determinants of effective disease management in Rheumatoid Arthritis to prevent persistently active disease: A qualitative study

Supplementary Material 4: Thematic map from interviews and focus group data

Main theme	*Sub-Theme - Facilitators*	*Sub-Theme - Barriers*	*Codes - Facilitators*	*Codes - Barriers*
**Life experiences and wellbeing practices**	• Mental and physical wellbeing practices	• Stressful life experiences and poor wellbeing	- Alternative treatments for pain reduction<br>- Maintaining hobbies and interests<br>- Physical wellbeing activities<br>- Positive thinking	- Balancing competing daily demands<br>- Mental stress<br>- Stressful event
**Socioeconomic (dis)advantages**	• Socioeconomic privileges	• Socioeconomic disadvantages	- Access to 'good quality' anti-inflammatory foods<br>- Access to private transport<br>- Availability of local green spaces<br>- Financial privileges<br>- Postcode lottery	- Cost of buying anti-inflammatory foods<br>- Cost of transportation<br>- Inadequate housing<br>- Local environment<br>- Structural issues in social care
**Employment and working conditions**	• Supportive employment and working conditions	• Issues with employment and working conditions	- Necessary work accommodations<br>- Participating in volunteering activities<br>- Supportive managers and colleagues	- Stress related to employment<br>- Unsupportive work
**Healthcare services**	• Effective treatment and care	• Adverse experiences of treatment and severity of illness	-Disability aids and adaptions<br>- Competent and friendly hospital staff<br>- Effective care<br>- Effective medication	-Increased severity of illness<br>-Medication issues<br>-Negative experience with healthcare professionals<br>-Structural challenges in healthcare
**Holistic RA information and knowledge**	• Health literacy and holistic knowledge	• Gaps in education and knowledge	-Holistic education about RA<br>- Useful and relevant medical education	- Lack of holistic education<br>- Misinformation about COVID-19

				- Misinformation about medications
**Social and familial support**	• Social and familial support	• Limited or no access to social and familial support	- Care and emotional support provided by family<br>- Religion and Spirituality<br>- Social media community<br>- Supportive friends	- Family and relationship issues<br>- Impaired social functioning<br>- Lack of disease awareness<br>- Loneliness
