# Supplementary material for: Understanding the psychosocial determinants of effective disease management in rheumatoid arthritis to prevent persistently active disease: a qualitative study
Source: RMD Open. 2024 Apr 12;10(2):e004104. doi: 10.1136/rmdopen-2024-004104 (PMC11029421; doi:10.1136/rmdopen-2024-004104)

Understanding the psychosocial determinants of effective disease management in Rheumatoid Arthritis to prevent persistently active disease: A qualitative study

Supplementary Material 5: Framework for the interplay between the psychosocial determinants of effective disease management and patient outcomes in Rheumatoid Arthritis, according to patients' perspectives.

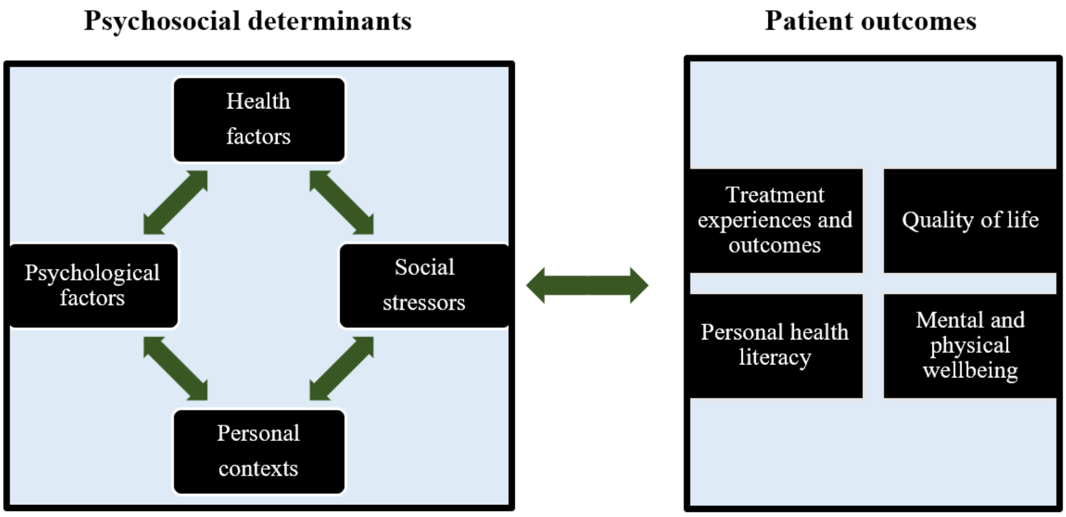

Supplement: Supplementary data [file rmdopen-2024-004104supp005.pdf]
