# Supplementary material for: Understanding the psychosocial determinants of effective disease management in rheumatoid arthritis to prevent persistently active disease: a qualitative study
Source: RMD Open. 2024 Apr 12;10(2):e004104. doi: 10.1136/rmdopen-2024-004104 (PMC11029421; doi:10.1136/rmdopen-2024-004104)
Supplement: Supplementary data [file rmdopen-2024-004104supp002.pdf]

Understanding the psychosocial determinants of effective disease management in Rheumatoid Arthritis to prevent persistently active disease: A qualitative study

Supplementary Material 2: Topic Guides

Focus Group Topic Guide

1. Introductions

- Please tell us your name and your preferred pronoun.

2. Icebreaker Questions

- A co-facilitator will ask each participant to briefly state their motivation for taking part in the focus group today.
- A co-facilitator will ask each participant what they hope to gain from taking part in the focus group today.

3. Free Listing Activity: Facilitators to Good Outcomes in Rheumatoid Arthritis

- Ask the participants to firstly think about the treatment of their Rheumatoid Arthritis, and then ask them to generate a list of factors that they feel have resulted in a good outcome of their Rheumatoid Arthritis.

Prompts:

- What is helpful in daily life for your treatment of Rheumatoid Arthritis (e.g., help to take or inject your medication in the right way)
- Help from your rheumatology consultant (other than the prescription of medication)
- Help from another healthcare professional (e.g., rheumatology nurse practitioner or pharmacist)
- Feeling sufficiently involved in the decision-making about treatment
- Support from family/friends
- Being at work, as well as a supportive work environment (e.g., colleagues, managers)
- Situations that help in the taking of medications
- The ability to self-manage your rheumatoid arthritis
- Being given adequate information about treatment, that has helped you make an informed choice/decision about treatment
- They feel that they have understood the information given about treatment
- Important goal or personal motivation to take or inject medication
- Beliefs about positive consequences of treatment (e.g., taking or injecting medication)

- Being signposted (e.g., to relevant members of the Multidisciplinary Team, for addressing other aspects of disease, e.g., comorbidities, physiotherapy, lifestyle [diet])

4. Discussion Question: Facilitators to Good Outcomes in Rheumatoid Arthritis

- Ask the participants to discuss the reasons why they have selected each of the factors that they feel have resulted in a good outcome of their Rheumatoid Arthritis.

5. Ranking Activity: Facilitators to Good Outcomes in Rheumatoid Arthritis

- Ask the participants to rank from top to bottom the factors that they chose in the free-listing activity that they feel have resulted in a good outcome of their Rheumatoid Arthritis.

- Ask the participants to explain the reasons why they have ranked the factors from top to bottom.

6. Free Listing Activity: Barriers to Good Outcomes in Rheumatoid Arthritis

- Ask the participants to firstly think about the treatment of their Rheumatoid Arthritis, and then ask them to generate a list of factors that have hindered a good outcome of their Rheumatoid Arthritis.

Prompts:

- Daily hassles in your life
- Barriers created by your rheumatology consultant (other than the prescription of medication)
- Barriers created by another healthcare professional (e.g., rheumatology nurse practitioner or pharmacist)
- Not feeling sufficiently involved in the decision-making about treatment
- Barriers created by family/friends
- Barriers created by the work environment (e.g., colleagues, managers)
- Situations that hinder the use of medications
- Not being able to self-manage your rheumatoid arthritis
- Not being given adequate information about treatment, which meant you could not make an informed choice/decision about treatment
- They feel that they have not understood the information given about treatment
- Important goal or personal motivation to not take or inject medication
- Beliefs about negative consequences of treatment (e.g., taking or injecting medication)
- Not being signposted, e.g., to relevant members of the Multidisciplinary Team, for addressing other aspects of disease, e.g., comorbidities, physiotherapy, lifestyle (diet)

7. Discussion Question: Barriers to Good Outcomes in Rheumatoid Arthritis

- Ask the participants to discuss the reasons why they have selected each of the factors that they feel have hindered a good outcome of their Rheumatoid Arthritis.

8. Ranking Activity: Barriers to Good Outcomes in Rheumatoid Arthritis

- Ask the participants to rank from top to bottom the factors that they chose in the free-listing activity that they feel have hindered a good outcome of their Rheumatoid Arthritis.

- Ask the participants to explain the reasons why they have ranked the factors from top to bottom.

9. Discussion Question: Factors that Contribute to Disease Activity

- What factors do you feel in your everyday life have made your Rheumatoid Arthritis more active.

Prompts:

- Daily hassles in your life
- Familial conflict
- Lack of social ties (e.g. friends)
- Work environment (colleagues, managers/pressure of job)
- Not being able to self-manage your rheumatoid arthritis
- Co-morbid conditions (e.g., mental health difficulties/other physical health conditions)
- Self-Isolation due to Covid-19 pandemic

- In what ways (if at all) do you feel that these factors have increased your level of inflammation.

10. Discussion Question: Participants' Perceptions about Successful Treatment of Rheumatoid Arthritis

- What influences your attendance to clinic for your Rheumatoid Arthritis?

Prompts:

- Daily hassles in your life
- (Positive or Negative) Relationship with Rheumatology Consultant

- The presence and tolerability of symptoms
- Feeling/not feeling sufficiently involved in the decision-making about treatment
- Un) Supportive family/friends
- (Un) Supportive work environment (e.g., colleagues, managers)
- (Not) being able to self-manage your rheumatoid arthritis
- Needle phobia (as a reason for minimising injections)
- Preference for Complementary and Alternative Medicine (distrust of synthetic treatment)
- Issues with commuting and transport to and from the Rheumatology Clinic
- Concerns about being immunocompromised during the Covid-19 pandemic

- What influences your adherence to treatment for your Rheumatoid Arthritis?

Prompts:

- Daily hassles in your life
- The presence and tolerability of symptoms
- Feeling/not feeling sufficiently involved in the decision-making about treatment
- (Un) Supportive family/friends
- (Un)Supportive work environment (e.g., colleagues, managers)
- Situations that hinder the use of medications
- (Not) Being able to self-manage your rheumatoid arthritis
- Being/not being given adequate information about treatment, that has helped you make an informed choice/decision about treatment
- They feel that they have (not) understood the information given to them about treatment
- Important goal or personal motivation to (not) take or inject medication
- Beliefs about positive/negative consequences of treatment (e.g., taking or injecting medication)
- Needle phobia (as a reason for minimising injections)
- Preference for Complementary and Alternative Medicine (distrust of synthetic treatment)
- Concerns about taking immunosuppressive treatment during the Covid-19 pandemic

- Thinking about what has resulted in or hindered a good outcome of your Rheumatoid Arthritis, what to you think is important for a patient with rheumatoid arthritis to be able to have successful treatment.

Prompts

- The role of the rheumatology consultant (other than the prescription of medication)
- Help by another healthcare professional (e.g., rheumatology nurse practitioner or pharmacist)

- Feeling sufficiently involved in the decision-making about treatment
- Support from family/friends
- Supportive work environment (e.g., colleagues, managers)
- Be given adequate information about treatment (e.g., what you would like to know about your medication, in order to take them or inject them)
- They feel that they have (not) understood the information given to them about treatment
- Important goal or personal motivation to take or inject medication
- Beliefs about positive consequences of treatment (e.g., taking or injecting medication)

11. End Focus Group Question

- End the focus group with the following question: Is there anything that we should have asked you that we did not ask you regarding what has resulted in or hindered a good outcome of your Rheumatoid Arthritis? (If the participants offer some suggestions – ask those questions)

12. Closing the Discussion

- Ask each participant for a closing statement on what has been discussed
- Summarise the main points of the discussion based on the co-facilitator's notes
- Debrief – ask participants if they have any comments or issues, they would like to raise
- Reiterate to participants that their names and comments will remain confidential
- Remind participants that they will each be sent an Amazon digital gift card voucher with a record of receipt form
- Thank participants for sharing their experiences with the group

BMJ Publishing Group Limited (BMJ) disclaims all liability and responsibility arising from any reliance placed on this supplemental material which has been supplied by the author(s)

Semi-Structured Interview Schedule

1. **What do you think needs to be present in someone's everyday life for them to be able to have successful treatment (e.g., successful outcome, complete disease control/remission, no symptoms – like pain, disability, absence of high disease activity, fatigue) for Rheumatoid Arthritis?**

Prompts

- Social ties/relationships/being able to socialise
- Support of family members (e.g., attitudes to the disease, material/practical/emotional support, help with family caring responsibilities – childcare, care of older relatives/relatives with disabilities)
- Employment if able to manage condition
- If employed, security of employment (e.g., secure full-time/part-time contracts vs agency, casual or zero-hour contracts)
- If employed, number of working hours (e.g., does not exceed 48 hours per week)
- If employed, supportive work environment (absence of/tackling of bullying/discrimination, implementation of reasonable adjustments)
- If employed, nature of work - demands/stressors of job
- Type of employment (e.g., manual vs non-manual/physically demanding vs non-physically demanding)
- Financial security (e.g., being able to afford a nutritious/low inflammation diet/food supplements/absence of financial debt)
- Adequate housing/accommodation
- Voluntary activities (e.g., participation in voluntary work)
- Religious faith/participation in religious activities/practices
- Absence of mental health stressors
- Management of any co-morbidities
- Access to private/public transport
- Access to adequate welfare/benefits support
- Level of education/educational background/educational understanding of rheumatoid arthritis
- The importance of education on rheumatoid arthritis and it's management for successful treatment
- The physical/social environment – feelings of safety, social networks and relationships with neighbours, accessible/inclusive physical environment for people with rheumatoid arthritis

2. What factors do you feel in your everyday life have helped you to manage (e.g., successful outcome, complete disease control/remission, no symptoms – like pain, disability, absence of high disease activity, fatigue) your Rheumatoid Arthritis?

Prompts

- Social ties/relationships/being able to socialise
- Support of family members (e.g., attitudes to the disease, material/practical/emotional support, help with family caring responsibilities – childcare, care of older relatives/relatives with disabilities)
- Being in employment or education
- If employed, security of employment (e.g., secure full-time/part-time contracts vs. agency, causal or zero-hour contracts)
- If employed, number of working hours
- If employed, supportive environment (absence of/tackling of bullying/discrimination, implementation of reasonable adjustments)
- If employed, nature of work - demands/stressors of work
- Type of employment (e.g., manual vs non-manual/physically demanding vs non-physically demanding)
- Financial security (e.g., being able to afford a nutritious/low inflammation diet/food supplements/absence of financial debt)
- Voluntary activities (e.g., participation in voluntary work)
- Religious faith/participation in religious activities/practices
- Absence of mental health stressors
- Management of any co-morbidities
- Access to private/public transport
- Access to adequate welfare/benefits support
- An understanding of rheumatoid arthritis and it's treatment
- Access to education on rheumatoid arthritis and its management for successful treatment
- The physical/social environment – feelings of safety, social networks and relationships with neighbours, accessible/inclusive physical environment for people with rheumatoid arthritis

3. In what ways do you feel that these factors in your everyday life have helped you to manage (e.g., successful outcome, complete disease control/remission, no symptoms – like pain, disability, absence of high disease activity, fatigue) your Rheumatoid Arthritis?

4. What aspects of your everyday life make your Rheumatoid Arthritis more active (e.g., symptoms of pain, fatigue, inflammation, disability, reduced mobility, flare ups, high disease activity)?

Prompts

- Social isolation/lack of social ties/lack of socialising
- Familial relationships (family members' attitudes to the disease, absence of material/practical/emotional support, social isolation from family members)
- If employed, unsecure employment (e.g., agency, causal or zero-hour contracts)
- Unemployment
- If employed, number of working hours (e.g., duration of working hours)
- If employed, unsupportive work environment (bullying/discrimination, non-implementation of reasonable adjustments)
- If employed, nature of work - demands/stressors of job
- Type of employment (e.g., manual vs non-manual/physically demanding vs non-physically demanding)
- Financial problems/uncertainty (stressors of indebtedness, not being able to afford a nutritious/low inflammation diet/food supplements)
- Co-morbid conditions (e.g., mental health difficulties/other physical health conditions)
- Housing/accommodation issues
- Problems with access to private/public transport
- Problems with accessing welfare/benefits support
- Not having a sufficient understanding of rheumatoid arthritis and its treatment
- Not being able to access education on rheumatoid arthritis and its management for successful treatment
- The physical/social environment – feelings of unsafety, lack of social networks and relationships with neighbours, feeling of disconnection from community life, inaccessible/non-inclusive physical environment for people with rheumatoid arthritis

5. **Can you describe any specific situation or incident from your everyday life that has made your Rheumatoid Arthritis more active (e.g., symptoms of pain, fatigue, inflammation, disability, reduced mobility, flare ups, high disease activity)?**

Prompts

- Disease activity duration (e.g., few hours, few days, or longer duration)
- Impact of disease (does the incident result in a proper disease flare or manageable pain)

6. **In what ways do you feel that these aspects of your everyday life have made your Rheumatoid Arthritis more active (e.g., symptoms of pain, fatigue, inflammation, disability, reduced mobility, flare ups, high disease activity)?**

Prompts

- Disease activity duration (e.g., few hours, few days, or longer duration)

- Impact of disease (does the incident result in a proper disease flare or manageable pan)

7. What factors in your everyday life help you to attend clinical appointments (e.g., with consultant rheumatologist, or for infusions) for your Rheumatoid Arthritis?

Prompts

- Support of family members (e.g., attitudes to the disease, material/practical/ support, help with family caring responsibilities – childcare, care of older relatives/relatives with disabilities)
- If employed, supportive work environment (implementation of reasonable adjustments, flexibility of employer to attend clinical appointments, employment conditions of workplace)
- Financial resources
- Access to private/public transport for appointments
- Communication between hospital/clinical department and patient
- Language support for patients with limited English proficiency (e.g., access to interpreters)
- Understanding/awareness of rheumatoid arthritis as a condition and its clinical treatment
- Received education on rheumatoid arthritis and its management for successful treatment

8. How do these factors in your everyday life help you to attend clinical appointments (e.g., with consultant rheumatologist, or for infusions) for your Rheumatoid Arthritis?

9. What factors in your everyday life present barriers to attending clinic appointments (e.g., with consultant rheumatologist, or for infusions) for your Rheumatoid Arthritis?

Prompts

- Lack of family support (e.g., attitudes to the disease, lack of material/practical support, lack of help with family caring responsibilities – childcare, care of older relatives/relatives with disabilities childcare)
- If employed, non-supportive work environment (non-implementation of reasonable adjustments, lack of flexibility of employer to attend clinical appointments, employment conditions of workplace)
- If employed, nature of work - demands/stressors of job

- Financial problems/uncertainty (e.g., transportation costs, can't take time off work due to financial costs)
- Lack of access to private/public transport for appointments
- Communication between hospital/clinical department and patient
- Language barriers for patients with limited English language proficiency (e.g., lack of access to interpreters)
- Preference for complementary and alternative medicine (distrust of synthetic medicine)
- Not having enough understanding/awareness of rheumatoid arthritis as a condition and its clinical treatment

10. Can you describe any specific situation or incident from your everyday life that has presented barriers to you for attending clinical appointments (e.g., with consultant rheumatologist, or for infusions) for your Rheumatoid Arthritis?

11. How do these barriers present problems for you in attending clinical appointments (e.g., with consultant rheumatologist, or for infusions) or your Rheumatoid Arthritis?

12. What factors in your everyday life help you to adhere to your treatment for the Rheumatoid Arthritis?

Prompts

- Support of family members (e.g., attitudes to the disease, material/practical/ support, help with family caring responsibilities – childcare, care of older relatives/relatives with disabilities childcare)
- If employed, supportive work environment (implementation of reasonable adjustments, flexibility of employer to attend clinical appointments for infusions)
- Access to private/public transport for clinical appointments for infusions
- Language support (e.g., access to interpreters) at clinical appointments for patients with limited English language proficiency
- Translated health information about treatments for patients with limited English language proficiency
- Understanding/awareness of rheumatoid arthritis as a condition and its clinical treatment
- Education on rheumatoid arthritis and it's management for successful treatment

13. How do these factors in your everyday life help you to adhere to your treatment for the Rheumatoid Arthritis?

14. What aspects of your everyday life prevent you from adhering to your treatment for the Rheumatoid Arthritis.

Prompts

- Lack of family support (e.g., attitudes to the disease, lack of material/practical support, lack of help with family caring responsibilities – childcare, care of older relatives/relatives with disabilities childcare)
- If employed, non-supportive work environment (non-implementation of reasonable adjustments, lack of flexibility of employer to attend clinical appointments for infusions)
- If employed, nature of work - demands/stressors of job
- Financial problems/uncertainty (e.g., transportation costs, can't take time off work due to financial costs to attend infusion appointments)
- Lack of access to private/public transport for clinical appointments for infusions
- Communication between hospital/clinical department and patient
- Lack of language support (e.g., access to interpreters) at clinical appointments for patients with limited English language proficiency
- Lack of translated health information about treatments for patients with limited English language proficiency
- Preference for complementary and alternative medicine (distrust of synthetic medicine)
- Not having enough understanding/awareness of rheumatoid arthritis as a condition and its clinical treatment
- Not being given education on rheumatoid arthritis and its management for successful treatment

15. Can you describe any specific situation or incident from your everyday life that has prevented you from adhering to your treatment?

16. How do these aspects of your everyday life prevent you from adhering to your treatment for the Rheumatoid Arthritis?

17. Is there anything else that you would like to comment on that you have not already mentioned in the interview, about how aspects of your everyday life affect the management and adherence to treatment for your rheumatoid arthritis?
